# When co-action eliminates the Simon effect: disentangling the impact of co-actor's presence and task sharing on joint-task performance

**DOI:** 10.3389/fpsyg.2013.00844

**Published:** 2013-11-19

**Authors:** Roberta Sellaro, Barbara Treccani, Sandro Rubichi, Roberto Cubelli

**Affiliations:** ^1^Interdepartmental Centre for Mind/Brain Sciences (CIMeC), University of TrentoTrento, Italy; ^2^Leiden Institute for Brain and Cognition, Institute for Psychological Research, Leiden UniversityLeiden, Netherlands; ^3^Department of History, Human Sciences and Education, University of SassariSassari, Italy; ^4^Department of Communication and Economics, University of Modena and Reggio EmiliaReggio Emilia, Italy; ^5^Department of Psychology and Cognitive Sciences, University of TrentoTrento, Italy

**Keywords:** spatial compatibility, joint Simon effect, joint action, social interaction, social cognition, division of labor, referential coding, action co-representation

## Abstract

This study aimed at assessing whether the mere belief of performing a task with another person, who is in charge of the complementary part of the task, is sufficient for the so-called joint Simon effect to occur. In all three experiments of the study, participants sat alone in a room and underwent two consecutive Go/NoGo tasks that were identical except for the instructions. In Experiment 1, participants performed the task first individually (baseline task), and then either co-acting with another person who responded from an unknown location to the NoGo stimuli (joint task) or imaging themselves responding to the NoGo stimuli (imaginative task). Relative to the baseline, the instructions of the imaginative task made the Simon effect occur, while those of the joint task were ineffective in eliciting the effect. This result suggests that sharing a task with a person who is known to be in charge of the complementary task, but is not physically present, is not sufficient to induce the representation of an alternative response able to produce interference, which happens instead when the instructions explicitly require to imagine such a response. Interestingly, we observed that when the Simon effect was already present in the baseline task (i.e., when the response alternative to the Go response was represented in the individual task due to non-social factors), it disappeared in the joint task. We propose that, when no information about the co-actor's position is available, the division of labor between the participant and co-actor allows participants to filter out the possible (incidental) representation of the alternative response from their task representation, thus eliminating potential sources of interference. This account is supported by the results of Experiments 2 and 3 and suggests that under certain circumstances task-sharing may reduce the interference produced by the irrelevant information, rather than increase it.

## Introduction

In the last years, a growing number of studies used the joint version of the Simon task to investigate whether and to what extent co-action (i.e., the execution of a task with another person) affects individual performance.

In a typical Simon task, the imperative stimulus (e.g., a red or green square) is presented on the left or right of the screen and participants have to press a left or right button depending on a non-spatial stimulus attribute (e.g., its color). Participants are usually faster and more accurate when the position of the stimulus corresponds to the position of the required response (corresponding trials) than when it does not correspond (non-corresponding trials). This effect (the so-called Simon effect; Simon and Small, 1969) is attributed to processes occurring at the response selection stage (e.g., Rubichi and Pellicano, 2004; Treccani et al., 2009). Basically, most accounts of the Simon effect assume that, even if irrelevant to the task, the stimulus position is automatically coded. The dimensional overlap between this task-irrelevant stimulus dimension and the response dimension (i.e., both dimension are spatial and refer to left and right) represents the prerequisite for either facilitation or interference processes to occur when the response is selected (see Kornblum and Lee, 1995). The irrelevant, automatically generated, spatial code of the stimulus is thought to interact with the spatial code of the response that is being activated on the basis of the task-relevant stimulus attribute. Response selection is facilitated when the stimulus and response codes are congruent: the selection of the required response benefits from the activation of a congruent spatial stimulus code. In contrast, response selection is interfered by an incongruent spatial code: when the two codes are incongruent, a conflict takes place that delays reaction times (RTs) or causes the selection of the incorrect response (see Wiegand and Wascher, 2005, and Proctor and Vu, 2006, for an overview of the main hypotheses advanced to account for the Simon effect).

The Simon effect is thought to be an index of the representation of two responses that are spatially alternative to one another. No spatial response coding, and thus no Simon effect, occurs when only one response, even though spatially denoted (e.g., the press of a button placed on the left-side of the participant), is involved, for example, in Go/NoGo tasks (Callan et al., 1974; Ansorge and Wühr, 2004, 2009). In two-choice tasks, indeed, spatial response coding has been shown to be based on response relative position: each response is coded with reference to the position of the alternative one (Umiltà and Nicoletti, 1985).

In the joint variant of the Simon task, two individuals, sitting next to one another, share the task in such a way that each person responds to only one of the two possible values of the stimulus by pressing a button in front of his/her body, that is, they perform two complementary Go/NoGo tasks (e.g., one participant responds to the red square only and the other participant responds to the green square only). Interestingly, a Simon effect is observed in these complementary Go/NoGo tasks: responses are faster when the position of the stimulus corresponds to the position of the responding participant with reference to the position of the co-actor (Sebanz et al., 2003; Tsai et al., 2006; see also Milanese et al., 2010, for an extension of the classical paradigm).

To account for the joint Simon effect, Sebanz et al. (2006) proposed the so-called *action co-representation account*. According to this account, in joint tasks participants tend to represent the co-actor's task (i.e., his/her aims and intentions: the response the co-actor is required to emit and the stimuli to which s/he has to respond), and integrate this representation in their action planning. As a consequence, participants' performance is impaired as if they were in charge of the co-actor's task as well. The spatial coding of two distinct responses leads to an interference effect (see Ferraro et al., 2011) when the participant's response is required but the position of the stimulus primes the co-actor's response. In this case, the spatial code of the stimulus does not correspond to the spatial code of the required response and this lengthens RTs similarly to what happens in standard (two-choice) Simon tasks.

Even though a number of studies have collected evidence in support of the action co-representation account of the joint Simon effect, this interpretation has been recently challenged by the results of other studies showing that the joint Simon effect might be mainly a spatial phenomenon rather than a social one (Guagnano et al., 2010; Dolk et al., 2011, 2013a; Dittrich et al., 2012, 2013).

For example, Guagnano et al. (2010) observed that two participants performing concurrently two independent detection tasks showed a Simon effect when they acted side-by-side, but not when they were far from each other. Guagnano et al. proposed that the joint Simon effect occurs because participants spatially code their own response using the position of the co-actor as a reference point. This reference point can only be used if the other person is located within the participant's peripersonal space. According to the authors, the representation of the co-actor's intentions would not have a functional role in the emergence of the Simon effect: the fact that another person is performing the task in close proximity simply leads the participant to code spatially his/her own response, which, in turn, provides the necessary conditions for the Simon effect to occur.

In the same vein, a recent study of Dolk et al. (2011) provided evidence against the action co-representation account. To test whether the joint Simon effect really reflects the integration of the co-actor's action into one's own action planning, the authors combined the joint Simon task with the so-called Rubber Hand Illusion (RHI; Botvinick and Cohen, 1998). The RHI is a well-known experimental manipulation that allows one to experience the illusion of ownership of a rubber hand when it is stroked synchronously with one's own hidden hand. On the contrary, the asynchronous stroking condition prevents the rubber hand to be included in one's own body schema. In contrast with what would have been expected on the basis of the action co-representation account (i.e., a larger joint Simon effect in the synchronous integrative condition than in the asynchronous non-integrative condition), the results showed the opposite pattern: the Simon effect was larger when participants perceived the co-actor's hand as separated from themselves (i.e., in the asynchronous stroking condition).

According to Dolk et al., the larger Simon effect observed in the asynchronous stroking condition is probably due to the fact that this manipulation emphasized the existence of an alternative action. On the basis of these results, Dolk et al. (2011) proposed the so-called *referential coding account* of the joint Simon effect (cf., Hommel, 1993). Following this account, the joint Simon effect it not really a social phenomenon but it occurs because the co-actor constitutes a salient event that provides participants with an alternative action, thus allowing them to code spatially their own response. Importantly, in a follow up study, Dolk et al. (2013a) demonstrated that any salient event able to attract attention (i.e., not necessarily a response emitted by another person but even, e.g., the movement of a ticking metronome) can represent an action alternative to that of the participant (i.e., an action from which the participant's response has to be discriminated). Consequently, the spatial coding of the participant's response position can occur with reference to any salient event. Interestingly, Vlainic et al. (2010) showed that such a spatial referential coding, once established, does not need online perceptual feed-back to be maintained: the joint Simon effect showed by two participants acting side-by-side continued to occur even when they are blindfolded. Further studies showed that spatial response coding can be based on different reference frames (see Dittrich et al., 2013). For instance, it may rely on the participant's position with reference to the co-actor's position (e.g., the participant's response may be coded as “left” if the co-actor is on the right side of the participant). Alternatively, spatial response coding can be based on the relative positions of the participant's and co-actor's response devices (e.g., regardless of the co-actor's position, the participant's response may be coded as “left” if the participant's response button is on the left with respect to the co-actor's response button), and participants may switch between the two reference frames (Dolk et al., 2013b; Liepelt et al., 2013; see also Milanese et al., 2011).

It is worth noting that both the action co-representation and the referential coding accounts share the assumption that in joint tasks the involvement of another person leads participants to represent an alternative action. Consistently, Sebanz et al. (2003) (Experiment 2) observed that no Simon effect occurs when the co-actor merely sat next to the participant, without emitting any response. In this case, indeed, there is no alternative action. The two accounts, however, differ from each other with respect to the nature of the joint Simon effect: social (for the action co-representation account) or spatial (for the referential coding account). Indeed, following the action co-representation account, the representation of the alternative action occurs because the participant represents the co-actor's task, which happens to be his/her complementary task (see Sebanz et al., 2005): the co-actor is responding to the alternative value of the stimulus with a spatially alternative response. A logical implication is that the mere knowledge about the co-actor's task, besides being necessary, should also be sufficient to give rise to the Simon effect. In contrast, the referential coding account states that an alternative action is represented simply by virtue of the fact that the co-actor's response constitutes a salient event, which cannot be ignored by the participant and from which the participant's action has to be discriminated, occurring on the side opposite to the participant's response position (Dolk et al., 2011, 2013a,b). As a consequence, the knowledge about the co-actor's task should be neither necessary nor sufficient for the Simon effect to occur.

Based on these premises, the problem of disentangling these two accounts can be turn into the problem of verifying whether the mere knowledge about the co-actor's task is sufficient to make participants represent an alternative response that produces interference, thus yielding the Simon effect.

A possible way to shed light on this question is to make the actor and co-actor execute their own part of the task in different rooms. If the belief of co-acting with another person who is responding to the complementary color is sufficient to code spatially the response associated with this color, the joint Simon effect should occur even when the co-actor performs his/her part of the task in a different, not-specified room. Conversely, if what allows participants to represent the alternative action is the fact that the co-actor represents a salient spatially-connoted event occurring next to the participant, no Simon effect should be observed when the co-actor is not physically present and executes his/her part of the task in a different room.

The present study aimed at evaluating the role of task-sharing in the occurrence of the joint Simon effect by eliminating the possible contribution of the *physical presence* of the co-actor. Studies that have previously tried to isolate the impacts of these two factors showed contrasting, and thus not conclusive results. Furthermore, both studies that have found the mere awareness of sharing a task with another to be sufficient to produce the joint Simon effect (Tsai et al., 2008 and Ruys and Aarts, 2010) and the study that has not (Welsh et al., 2007) involved possible confounding factors that might account for their results. The present study was designed in order to control for these factors and solve such inconsistencies.

The first study that explicitly addressed this issue is the work by Welsh et al. (2007). These authors failed to observe the joint Simon effect when the co-actor was thought to perform the complementary task in a different room. However, Welsh et al. used a within-participants design in which participants performed four tasks in a fixed order (i.e., the two-choice task, the individual Go/NoGo task, the co-actor present joint task and the co-actor absent joint task). Therefore, one cannot rule out that the lack of a significant Simon effect in the forth critical task (i.e., the joint task in which the co-actor was absent) is due to practice effects. Indeed, even though the Simon effect has been proved to be robust, it can be significantly reduced through practice (e.g., Simon et al., 1973).

In contrast with Welsh et al. (2007), two recent studies showed that the Simon effect can be observed in joint tasks even when the actor and the co-actor performed their parts of the task in two different rooms (Tsai et al., 2008; Ruys and Aarts, 2010), thus suggesting that the mere belief of co-acting with another person can be sufficient to induce the joint Simon effect. However, some methodological concerns of these two studies do not allow ascribing unequivocally the occurrence of the Simon effect to the social manipulation.

In Tsai et al.'s (2008) study, participants sat alone in a room and were instructed to press the right button of a computer mouse when a lateralized green square appeared on the screen and to refrain from responding when a red square was presented. They were told that they would have performed the task with another individual, who was in another room and responded to the complementary color (the red square) by pressing the left button. A Simon effect was observed: responses were faster when the target position corresponded to the participant's response button (i.e., the target was on the right) than when it did not correspond (i.e., the target was on the left).

In line with the action co-representation account, the Simon effect observed by Tsai et al. (2008) could be explained by assuming that the belief of co-acting with another individual, responsible for the complementary color, let participants to activate, not only the representation of the action they had to execute, but also the representation of the co-actor's action. Yet, this effect could be traced back to spatial (non-social) factors rather than to the knowledge about the co-actor's task. That is, the representation of both left and right responses might have been prompted by the use of the mouse as response devise.

It is well-known that, although the Simon effect does not usually occur in individual Go/NoGo tasks (Callan et al., 1974), there are several exceptions to this rule: the Simon effect may be observed in this kind of tasks when the experimental conditions lead participants, not only to activate the required response, but also to code another (non-operative) response. This occurs, for example, when a Go/NoGo task is preceded by a two-choice task in which participants used two response buttons (Ansorge and Wühr, 2004, 2009; see also Lugli et al., 2013). As previously discussed, the occurrence of the Simon effect is reckoned to be an index of the representation of two spatially alternative actions. Accordingly, in these Go/NoGo tasks, participants are thought to represent both the Go response and the alternative not-required response. That, in turn, is attributed to their previous experience with the two-choice task, which required the actual execution of the alternative response: the representation of the alternative response, activated in the two-choice task, is transferred to the subsequent (Go/NoGo) task, even though in the second task this response is no longer task relevant.

In Tsai et al.'s (2008) study the participant did not perform a two-choice task before the critical joint task. However, prior experience might have been crucial for the occurrence of the Simon effect in their paradigm as well. The response device used in Tsai et al. involves two possible responses: the standard computer mouse presents two response buttons and is made in such a way that, in order to operate it effectively, both the index and middle fingers have to be placed on the corresponding buttons even when only one button has to be pressed. These two responses are not comparable in terms of frequency of use: the left button (i.e., the primary mouse button in standard multi-button mice) is more frequently used than the right one. All participants of Tsai et al.'s study responded to the Go stimuli by pressing the (secondary) right mouse button. Similarly to what happens when the Go/NoGo task is preceded by a task that involves the alternative response, the practice in daily life with the left mouse button might have led participants of Tsai et al.'s study to activate the representation of the left button in addition to that of the right one (i.e., when using the secondary mouse button, participants could not help coding the primary one as well). The resulting spatial response coding might have been the cause of the observed Simon effect. Obviously, a similar Simon effect is not at all social in nature: it would have occurred even if participants had been told that they were performing the task by themselves. Tsai et al.'s did not control for the possible role of such factors in the occurrence of the Simon effect and did not provide for a control condition in which participants believed that they were on their own while performing the Go/NoGo task. Accordingly, the occurrence of the Simon effect in their joint task cannot be unequivocally ascribed to the belief of co-acting with another person and to the representation of the response emitted by this person. It is also worth noting that the position of the co-actor's response was not the only spatial cue with which participants were provided in Tsai et al.'s study. They also knew the position of the room in which the co-actor was acting: the co-actor was thought to perform the task in an adjacent room that had been shown to the participant before the experimental phase. This might have had a role in inducing the spatial effect observed in Tsai et al.'s joint task.

Unwanted spatial factors involved by the experimental procedure can also account for the joint Simon effect observed by Ruys and Aarts (2010). In this study, participants performed an auditory version of the joint Simon task: they were instructed to respond to a certain tone by pressing a right-side key (“3” on the numerical keyboard) and to withhold the response to another tone because another person, who was in another room, responded to it by pressing a left-side key (“z”). The joint Simon effect was observed although the co-actor was acting in a different room: participants were faster when the Go tone was presented at the right ear than when it was presented at the left ear. However, the occurrence of the Simon effect can be justified by a minor detail of the experimental procedure: in order to remind participants that another person was engaged in the task, the co-actor's responses were signaled with a red light occurring on the left side of the screen, whereas participants' responses were signaled with a red light occurring on the right side. Thus, even if the co-actor was not physically present, the lateralized light, spatially corresponding to the response button of the co-actor, represents a salient, visible event, which stands for an action alternative to that of the participant. The fact that the responses of both the participant and the co-actor were followed by the presentation of lateralized lights, spatially corresponding to their respective response buttons, might have induced participants to code their own response as “right” as opposed to the left light signaling the co-actor's response. Furthermore, given that the participant and the co-actor were recruited in couples and placed in adjacent rooms, very likely they both knew their relative positions, as in Tsai et al.'s (2008) study.

Thus, whether the mere knowledge about the co-actor's task is sufficient to give rise to the Simon effect remains to be ascertained. In order to address this issue and to control for the possible role of other (non-social) factors in the occurrence of the Simon effect in prior studies (e.g., Tsai et al., 2008), we decided to employ a paradigm that, despite being similar to that of Tsai et al., allowed us to test the effects of both task-sharing and the device used to respond (a computer mouse) on the representation of an action alternative to the participant's response. These effects were intended to be compared with those observed when the representation of such an alternative action was explicitly requested by the task instructions.

In all the experiments of the present study, participants sat alone in a room and were required to respond to the target stimuli by pressing one of the two buttons of the mouse. Although only one response was requested, the response device involved a possible alternative response (i.e. the press of the non-requested mouse button). In Experiment 1, we manipulated the task instructions to compare two critical experimental conditions. In one of them, participants were explicitly required to activate the representation of the alternative response: they had to imagine that they were responding to the alternative stimulus with the alternative response^1^. In the other condition, participants were simply told that another person was responding (from an unknown location) to the alternative stimulus.

Contrary to Tsai et al. (2008), we counterbalanced across participants the mouse button used to respond. As mentioned above, the two mouse buttons are not comparable in terms of frequency of use, and this might cause different spatial effects for participants who used the left and right mouse buttons. Such findings would have extended the implications of studies on between-task transfer of spatial response representations (Ansorge and Wühr, 2004, 2009; Lugli et al., 2013). Therefore, this study could not only help to clarify the inconsistencies between the results of prior studies on the joint Simon effect (e.g., Welsh et al., 2007; Tsai et al., 2008), but also provide cues for the comprehension of the impact of previous experience in standard (individual) Simon tasks.

## Experiment 1

Experiment 1 aimed at testing the predictions of the two main accounts of the joint Simon effect (i.e., the action co-representation account, Sebanz et al., 2006, and the referential coding account, Dolk et al., 2013a) in some critical conditions meant to isolate the factors that these accounts indicate as crucial for the occurrence of the effect. First of all, participants performed an individual Go/NoGo task (i.e., the baseline task), which required them to press one of the two buttons of the mouse when the target stimulus was of one of two alternative colors. Afterwards, they executed a second task (i.e., either an imaginative two-choice task or a joint task) which was identical to the baseline task except for the instructions. In the imaginative two-choice task, besides responding to the Go color, participants were also asked to imagine themselves responding to the NoGo color by pressing the alternative mouse button. In the joint task, participants continued to respond to the Go color but they also believed that they were performing the task with a co-actor, who was in a non-specified room and was responding to complementary (NoGo) color. Actually, participants performed the task on their own. Finally, to control for possible practice effects, a third group of participants was required to continue to perform the Go/NoGo task individually.

Given that transfer effects from one task to another are very common in this kind of tasks (Ansorge and Wühr, 2004; Lugli et al., 2013; Ansorge and Wühr, 2009), we preferred not to counterbalance the order of the two tasks. Therefore, all participants started with the individual Go/NoGo task, enabling us to consider this individual task as a proper baseline.

The following predictions could be made. First of all, as mentioned above, we expected to observe a Simon effect in the baseline task, but only for participants who responded to the Go stimuli by pressing the right mouse button (i.e., the less frequently used mouse button). No Simon effect should emerge for participants using the left mouse button.

In the imaginative two-choice task, which explicitly required participants to activate the representation of the NoGo response, a Simon effect was expected, regardless of the mouse button used to respond.

In the joint task, different results were expected on the basis of the two main hypotheses advanced to explain the joint Simon effect. The action co-representation account states that the belief of co-acting with another person responsible for the complementary color is sufficient to induce participants to represent the co-actor's response and to integrate it in their action planning (Sebanz et al., 2005, 2006; Tsai et al., 2008). Accordingly, both left- and right-button participants should show the joint Simon effect, at least when they knew that the co-actor responded with the alternative response button. Thus, these participants should show the same pattern of results as that shown by participants who performed the imaginative two-choice task. Conversely, according to the referential coding account, the joint Simon effect only depends on the co-actor constituting a salient, spatially-connoted action event that occurs next to the participant (i.e., an attention-attracting, dynamic event, which cannot be ignored and from which the participant's response has to be spatially discriminated; Guagnano et al., 2010; Dolk et al., 2011, 2013a). Only such an event may represent a reference point for the spatial coding of the participants' response. Following this account, no spatial response coding, and thus no Simon effect, could be induced by the co-actor in the joint task used here: in this task, the co-actor was not physically present and neither were there other perceivable stimuli in the experimental setting that attracted attention and could stand for the co-actor's action (cf., Ruys and Aarts, 2010). Therefore, the Simon effect might be present in the joint task only for those participants who had already (and irrespective of the belief of co-acting with another individual) activated the alternative response and coded the position of the requested response, that is, right-button participants.

### Methods

#### Participants and experimental design

Sixty-four undergraduate students of the University of Trento (9 males; aged 19–31 years; all right-handed) participated in the experiment. All participants were naïve about the purpose of the experiment and had normal or correct-to-normal vision.

**Figure 1:**
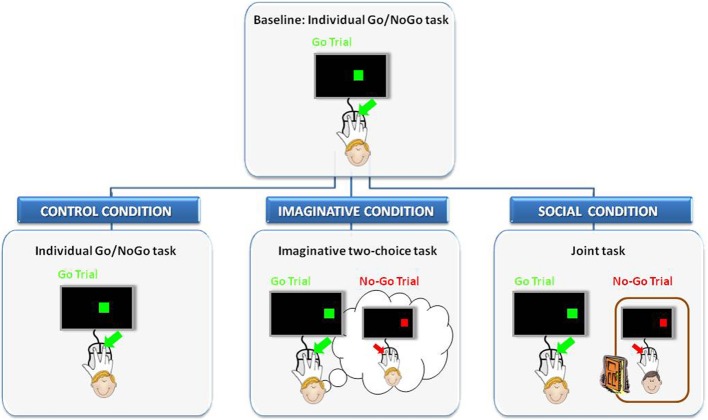
**Schematic representation of the experimental design adopted in Experiment 1.** Participants were randomly assigned to one of three experimental conditions: control, imaginative or social. Each experimental condition comprised two tasks (i.e., the baseline individual task and the critical task). Participants assigned to the control condition, after completed the baseline, simply continued to perform the same Go/NoGo task individually. Participants assigned to the imaginative condition, once completed the baseline, performed a task requiring them to imagine responding to the complementary color. In contrast, participants assigned to the social condition, after the baseline, were required to perform the same Go/NoGo task with an alleged co-actor who was in a non-specified room and was thought to respond to the complementary color.

Participants were randomly assigned to one of three experimental conditions: the control, imaginative, or social condition. Sixteen participants were assigned to either the control or the imaginative condition, whereas 32 participants were assigned to the social condition. Each condition comprised two tasks: the baseline task and one of three critical tasks. Conditions were defined as control, imaginative, or social depending on the task that participants performed after the baseline: the repetition of the individual Go/NoGo task, the imaginative two-choice task and the joint task, respectively (see Figure 1).

#### Apparatus, stimuli, and procedure

All participants seated about 57 cm from a 17-inch monitor screen and performed two tasks, with a 5-min break in between.

In all tasks, participants were instructed to press one of the two buttons of a computer mouse in response to stimuli of one color (Go color) and not to respond to stimuli of the other color (NoGo color). The mouse was aligned with the middle of the screen. Half of the participants responded with the left button, which was operated with the index finger of the right hand; the other half responded with the right button, which was operated with the middle finger of the right hand. The Go color and the response position were counterbalanced across participants and were kept constant during the experiment (i.e., they were the same in the two consecutive tasks). In the imaginative two-choice task, participants were also asked to imagine responding to the NoGo color, whereas in the joint task they believed that another person was responding to the NoGo color. In the latter task, half of the participants were told that the co-actor was responding with the same button as them, whereas the other half were told that the co-actor was using the alternative button.

In all tasks, trials began with presentation of a central 0.8^°^ × 0.8^°^ white fixation cross, which remained visible for 500 ms. At the offset of fixation, the target stimulus (i.e., a 1.9^°^ × 1.9^°^ colored square) was presented for 300 ms. The target was shown either on the left or on the right of the fixation cross (the center of the target was horizontally aligned with the fixation cross, 5.7^°^ to the left or right) and it could be either green or red. Both the fixation cross and target were presented on a black background. Offset of the target was followed by a 500-ms blank interval. Thus, on the whole the time allowed for the response was 800 ms. Missed responses, responses with latencies in excess of 800 ms, responses to stimuli of the NoGo color and responses with the wrong button were all counted as errors. If the response was correct, the trial terminated with an additional 400-ms blank interval. In the case of an error, a 200-ms visual error feedback (a string of six exclamation marks) was presented instead, followed by a 200 ms blank interval.

In the joint task, a *click* sound, randomly ranging from 240–622 ms, was delivered during the NoGo trials. It was emanated from two loudspeakers that were contiguous to the two sides of the monitor screen (one on the left and one on the right). This sound signaled the co-actor's response and was meant to increase the participant's belief that another person was engaged in the task. At the beginning of each block of joint-task trials, participants were instructed to press their response mouse button to inform the alleged co-actor that they were ready to start. Afterwards, a *click* sound and the presentation of an “ok” message on the screen signaled to the participant that the co-actor was ready to start as well. The computer delivered the co-actor's reply after a random time interval.

Each task consisted of 240 randomly mixed trials divided into two blocks. There were 120 Go trials and 120 NoGo trials. In half of the Go trials, stimulus and response positions corresponded, whereas, in the other half, stimulus and response positions did not correspond. Experimental trials were preceded by 8 practice trials.

### Results

Error data (0.8%) were not analyzed. We first analyzed RTs of the baseline task. Correct mean RTs were submitted to an analysis of variance (ANOVA) with spatial correspondence (corresponding vs. non-corresponding) as within-subjects factor and two between-subjects factors: response button position (left- vs. right-button participants) and condition (imaginative, social, and control).

The analysis revealed a significant main effect of correspondence [*F*_(1, 58)_ = 21.97, *p* < 0.001; η^2^_p_ = 0.28]. Participants were faster in corresponding than in non-corresponding trials (332 vs. 340 ms). Response position was also significant [*F*_(1, 58)_ = 4.13, *p* < 0.05; η^2^_p_ = 0.07]: left-button participants were faster than right-button participants (325 vs. 346 ms). More important, a significant Response position × Correspondence interaction was found [*F*_(1, 58)_ = 10.94, *p* < 0.01; η^2^_p_ = 0.16]. *Post-hoc* analysis (Newman–Keuls) revealed that responses of right-button participants were faster in corresponding than non-corresponding trials (340 vs. 353 ms, *p* < 0.001), whereas the two types of trials did not differ from each other for left-button participants (324 vs. 326 ms, *p* = 0.56). Condition did not yield a significant main effect and did not interact with any other factors.

**Figure 2:**
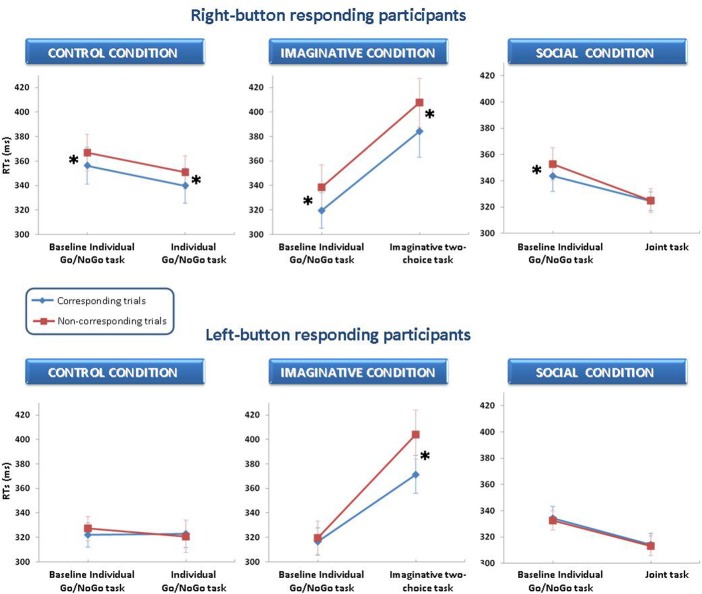
**Means (± S.E.M.s) of correct RTs for each experimental condition in Experiment 1.** For each condition, RTs are plotted as a function of response button position (left- vs. right-button participants), task (baseline vs. second task) and spatial correspondence (corresponding vs. non-corresponding trials). Asterisks indicate the presence of the Simon effect.

Next, we compared the baseline and the critical tasks of each condition (see Figure 2).

Given the differences between left- and right-button participants observed in the baseline task, separate ANOVAs were conducted for the two response button positions. In each ANOVA, there were two within-subjects factors: task (baseline vs. critical task) and correspondence (corresponding vs. non-corresponding). In the social condition, besides the two within-subjects factors, the co-actor's response button (same- vs. alternative-button) was included in the analysis as between-subjects factor.

The main effect of task was significant in both imaginative and social conditions and for both left- and right-button participants (all *F*s ≥ 7.24; all *p*s < 0.05, all η^2^_p_s ≥ 0.34), whereas it was not significant for either group of participants in the control condition. In the imaginative condition, participants were slower in the imaginative two-choice than in the baseline task (388 vs. 318 ms and 396 vs. 329 ms, for left and right-button participants, respectively). In the social condition, participants were faster in the joint than in the baseline task (314 vs. 333 ms and 325 vs. 348 ms, for left and right-button participants, respectively).

For right-button participants, a significant main effect of correspondence was observed in all experimental conditions (all *Fs ≥ 6.28*, all *p*s < 0.05, all η^2^_p_s ≥ 0.31). In contrast, for left-button participants, correspondence had a significant main effect only in the imaginative condition [*F*_(1, 7)_ = 12.62, *p* < 0.01; η^2^_p_ = 0.64].

In the imaginative condition, the Task × Correspondence interaction was significant only for left-button participants [*F*_(1, 7)_ = 14.84, *p* < 0.01; η^2^_p_ = 0.68]. These participants showed no Simon effect in the baseline task (*p* = 0.59), whereas they showed a 32-ms Simon effect in the imaginative two-choice task (*p* < 0.001). In contrast, for right-button participants, the interaction was not significant: the Simon effect was present in both tasks (19- and 23-ms in the baseline and in the imaginative two-choice tasks, respectively).

In the social condition, we found the opposite pattern of results. The Task × Correspondence interaction was significant only for right-button participants [*F*_(1, 14)_ = 4.61, *p* < 0.05; η^2^_p_ = 0.25]. For these participants, corresponding trials yielded faster responses than non-corresponding trials in the baseline task (343 vs. 353 ms; *p* < 0.001), whereas there was no difference between corresponding and non-corresponding trials in the joint task (324 vs. 325 ms; *p* = 0.88). Conversely, for left-button responding participants, this interaction was not significant. The Simon effect was, indeed, absent in both the baseline and joint tasks. Importantly, the three-way interaction was significant for neither group of participants, which indicates that the same pattern of results was obtained regardless of the co-actor's button. Indeed, the co-actor's button did not have a significant main effect either, and none of the interactions involving this factor was significant. The Simon effect shown by right-button participants in the baseline task disappeared in the joint task, whereas left-button participants continued to shown no correspondence effects, irrespective of whether the co-actor was thought to use the same button as the participant or the alternative button: the non-corresponding–corresponding differences in the same- vs. alternative-button conditions of the joint tasks were 1 vs. 0 ms for right-button participants and 1 vs. −3 ms for left-button participants.

In the control condition, the Task × Correspondence interaction was significant for neither right- nor left-button participants. Right-button participants showed an 11-ms Simon effect both in the baseline task and in the repetition of the individual Go/NoGo task, whereas left-button participants showed no effect in either task.

### Discussion

The results obtained in the baseline task showed no Simon effect for left-button participants: consistent with the results of previous studies (e.g., Ansorge and Wühr, 2004), the Simon effect did not occur in an individual Go/NoGo task in which participants only represented the Go response. The performance of these participants thus represented a pure baseline to test whether the mere belief of co-acting with another person who responds to the complementary color is sufficient to give rise to the Simon effect. In contrast, right-button participants showed a significant Simon effect. This finding confirms our predictions and suggests that right-button participants also represented the alternative (left) button, although it was task irrelevant and it was not mentioned in the instructions. Despite performing a Go/NoGo task, these participants spatially code two alternative responses, which provides the necessary conditions for the Simon effect to occur (i.e., a stimulus-response spatial dimensional overlap; Kornblum and Lee, 1995). In accordance with this account, right-button participants were found to be slower than left-button participants, who presumably represented only the required response, thus preventing possible competitions between responses from slowing down RTs (i.e., right-button RTs may involve an additional processing stage—response selection—which is actually not required by a Go/NoGo task; cf., Donders, 1868/1969). These results are consistent with previous findings demonstrating the role of both contextual factors and previous experience (here, the massive practice with the left mouse button in daily life) in determining the representation of two alternative responses in individual Go/NoGo tasks (Ansorge and Wühr, 2004, 2009; Lugli et al., 2013). In the first place, therefore, the present findings make a considerable contribution to the literature concerning the occurrence of Simon effects in Go/NoGo tasks. They show that the response device itself may be critical in this kind of task: it can induce participants to represent and code spatially an alternative (non-requested) response, thus giving rise to the Simon effect (cf., Dittrich et al., 2012).

More interestingly, participants' performance was critically modulated by the kind of task executed after the baseline. Notably, the instructions of the imaginative two-choice task and those of the joint task gave rise to opposite effects. First of all, they had a different impact on participants' response speed. Regardless of the response button used to respond, RTs slowed down in the imaginative two-choice task compared to the baseline task, whereas the opposite trend was observed in the joint task. The significant increase of RTs in the imaginative two-choice task is consistent with previous findings showing that the mental simulation of an action is functionally similar to its actual execution (e.g., Decety et al., 1989; Decety and Grèzes, 2006). Thus, it is reasonable to reckon that the imaginative two-choice task was similar to the standard two-choice Simon task: in both cases the representation of two alternative and competitive responses slows down RTs compared to tasks providing for only one possible response (i.e., standard Go/NoGo tasks; Donders, 1868/1969)^2^. Conversely, in the joint task a significant reduction of RTs was observed. That is consistent with the findings of previous studies (e.g., Sebanz et al., 2003) and is thought to be due to an increment of the arousal induced by the mere presence of another person while performing the task (i.e., the so-called social facilitation effect; Guerin, 1993; Aiello and Douthitt, 2001). The social facilitation effect observed here suggests that, although participants never met the alleged partner, our social manipulation was effective in inducing the belief of co-acting with another person.

More importantly for the purpose of this study, and contrarily to the predictions of the action co-representation account, the manipulations involved in the imaginative two-choice and joint tasks had opposite influences on the occurrence of the Simon effect.

In the imaginative two-choice task, the explicit request of representing the alternative response induced participants either to activate this representation (for left-button participants) or to keep it active (for right-button participants). That made the Simon effect emerge even when it had not occurred in the baseline task. Left-button participants—who did not exhibit any effect in the baseline task—showed a significant Simon effect in the imaginative task, whereas right-button participants continued to show the same (significant) effect shown in the baseline task.

In contrast, in the joint task, the belief of co-acting with an unseen co-actor was ineffective in eliciting the Simon effect; quite the opposite, such a belief seems to have been effective in eliminating the effect when it was present in the individual (baseline) condition. Left-button participants continued to show no difference between the two correspondence conditions, whereas for right-button participants the effect observed in the baseline task disappeared.

The absence of the Simon effect in the joint task (irrespective of the button used by the co-actor) is conflicting with the assumption—underlying the action co-representation account—that sharing a Simon task with a co-actor, responsible for the complementary target color, leads participants to activate the representation of the response associated with this color (the co-actor's action). Based on this account, indeed, the mere belief of co-acting with another individual, who responds to the complementary color with an alternative response, should give rise to the Simon effect just like the explicit request to activate the representation of the alternative response. On the contrary, the absence of any spatial correspondence effect in the joint task is in line with the referential coding account of the joint Simon effect: it shows that the mere belief of co-acting with another person and the mere knowledge about the co-actor's task are not sufficient to make participants represent the alternative action. When the co-actor is not physically present, the joint Simon effect does not show up.

Apart from the ineffectiveness of the joint task in inducing the Simon effect, another aspect of our results deserves attention: the joint instructions made the Simon effect disappear for the right-button participants who showed it in the baseline task. This result is particularly interesting because, given that transfer effects from one task to the other are very common (Ansorge and Wühr, 2004, 2009; Lugli et al., 2013), one would have expected to keep observing the Simon effect in this group of participants when they performed the joint task.

The disappearance of the Simon effect cannot be traced back to practice effects, as demonstrated by the results obtained in the control condition. Indeed, right-button participants continued to show the Simon effect in the repetition of the Go/NoGo task. It is worth noting that these findings provide additional evidence of the effectiveness of the social manipulation: if the social manipulation had been ineffective in making participants believe that another person was involved in the task, it should obviously have had no effects on performance and, in the second (joint) task, right-button participants of the social condition should have continued to show the Simon effect shown in the first (baseline) task, as the participants of the control condition who simply repeated the task individually.

The disappearance of the Simon effect can be accounted for by assuming that in the joint task a division of labor between the participant and the co-actor was established so as to allow right-button participants to filter out the representation of the alternative (left) response from their task representation. It is plausible that, when performing the baseline task, right-button participants associated the left response, which they had represented although it was not task-relevant, with the complementary color. In the joint task, however, due to the belief that the actor was responding to the complementary color, these participants might have attributed this alternative response (as well as the complementary color) to the co-actor. Given that these participants had no clue about the position of the co-actor, the response associated with the co-actor might have lost its spatial connotation. As a consequence, only one response remained active in participants' task representations and reference points for spatial response coding were no longer available, which resulted in the disappearance of the Simon effect. When only a non-spatial response is represented, no overlaps between stimulus and response dimensions (and no matches/mismatches between stimulus and response codes) occur that can produce the Simon effect (Kornblum and Lee, 1995).

It is important to underline that such a division of labor seems not to imply at all the representation of the co-actor's response, as indicated by the fact that the Simon effect shown by right-button participants disappeared in the joint task irrespective of whether they thought that the co-actor was responding with the same mouse button as them or with the alternative one. It is simply the fact that the co-actor was in charge of the NoGo color that seems to be critical in removing the response that was associated with this color in participant's task representation. This would happen regardless of the response button used by the co-actor to take care of such NoGo stimuli.

On the contrary, whether or not the participants know the position of the co-actor might be relevant for the effectiveness of the division of labor. Tsai et al. (2008) found a significant Simon effect for participants who responded with the right mouse button in a task wherein the co-actor was not physically present and was thought to be in charge of the NoGo color. Such an experimental condition should lead to the division of labor between the participant and the co-actor. Yet, in Tsai et al.'s study participants were aware of the position of the co-actor's room. In contrast, no information about the co-actor's position was provided here. As mentioned above, it is plausible that the division of labor was effective in making the Simon effect disappear because participants did not know the position of the person to which they associated both the NoGo stimuli and the alternative response. This might have been critical in letting the alternative response, which was represented although not-required, lose its spatial connotation and its role as reference point for the spatial coding of the required response.

If this were the case, then the Simon effect should persist both when the co-actor is thought to respond to the same color as the participant (i.e., when there is no division of labor) and when the co-actor is supposed to work on the complementary color but the participant knows where the co-actor is. These possibilities were tested in Experiments 2 and 3, respectively.

## Experiment 2

The disappearance of the Simon effect observed in the joint task of Experiment 1 is ascribable to a division of labor between the participant and the co-actor: knowing that another person (the co-actor) was in charge of the complementary color, right-button participants might be induced to attribute the alternative response, which they had previously associated with this color, to the co-actor. That would have allowed them to filter out the alternative response from their task representation, as no spatial information about the co-actor's position was available. If this were true, right-button participants should continue to show the Simon effect in the joint task when task instructions hamper them from attributing the alternative response to the co-actor. Experiment 2 aimed at testing this hypothesis. To this end, after completing the baseline task, participants executed a joint task identical to that of Experiment 1 except that they were told that the co-actor, who was in a not-specified room, was responding to their same Go color (e.g., both the participant and the co-actor responded to the green color). The following predictions could be made. First of all, we expected to replicate the pattern of results observed in the baseline tasks of Experiment 1: only right-button participants should show the Simon effect in the individual Go/NoGo (baseline) task, whereas left-button participants should not.

In the joint task, the Simon effect was not expected to occur for left-button participants. Indeed, the joint Simon effect has been proved not to occur even when the co-actor is present if the co-actor is thought to respond to the same target color as the participant (see Lam and Chua, 2010). In contrast, we expected right-button participants to keep showing a Simon effect in the joint task: given that the co-actor is thought to respond to the same color as the participant, the alternative (left) response cannot be attributed to anyone else and it should remain active in participants' task representation.

### Methods

#### Participants

Thirty-two undergraduate students of the University of Trento (5 males; aged 19–27 years; all right-handed) participated in the experiment.

Participants were not aware of the purpose of the experiment. They did not participate in the previous experiment and had normal or correct-to-normal vision.

#### Apparatus, stimuli, and procedure

The apparatus, stimuli and procedure were as in the social condition of Experiment 1 with the following exceptions. After the baseline task, participants performed a joint task in which they believed that the co-actor was performing their same task (i.e., s/he was responding to their same Go stimuli). The *click* sound signaling the co-actor's response was delivered at the end of the Go trials, 800 ms after the presentation of the Go stimulus, thus avoiding possible overlaps with the participant's response. Participants were told that the *click* sound did not correspond in time to the co-actor's response and that it was only meant to inform them whether the co-actor had responded or not.

### Results and discussion

Error data (0.5%) were not analyzed. Correct mean RTs of left- and right-button participants were submitted to two ANOVAs with the same factors of the social condition of Experiment 1. Again, the co-actor's response button (same- vs. alternative-button) did not yield any significant effects.

The effect of task was significant for both left and right-button participants (both *F*s ≥ 7.02, both *p*s < 0.05, both η^2^_p_ ≥ 0.33). Consistent with the pattern of results observed in the social condition of Experiment 1, RTs were faster in the joint than in the baseline task (331 vs. 340 ms and 321 vs. 340 ms, for left- and right-button participants, respectively).

The effect of correspondence was significant only for right-button participants [*F*_(1, 14)_ = 38.34, *p* < 0.001; η^2^_p_ = 0.73]: responses were faster in corresponding than non-corresponding trials (324 vs. 337 ms).

**Figure 3:**
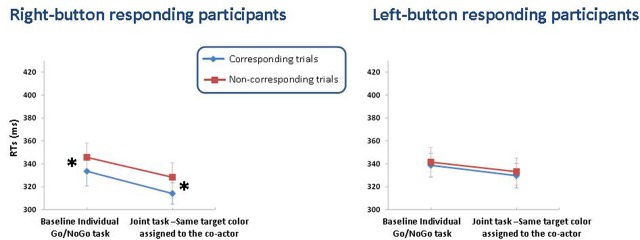
**Means (± S.E.M.s) of correct RTs for right- and left-button responding participants in Experiment 2.** RTs are plotted as a function of task (baseline vs. joint task) and spatial correspondence (corresponding vs. non-corresponding trials), separately for the two response button positions (right- vs. left-button participants). Asterisks indicate the presence of the Simon effect.

The Task × Correspondence interaction was significant for neither group of participants (see Figure 3).

The results of Experiment 2 confirmed the predictions. Left-button participants did not exhibit the Simon effect in either the baseline or joint task. Conversely, right-button participants showed a Simon effect in both tasks: corresponding trial advantages of 12 and 14 ms were observed in the baseline and joint tasks, respectively.

Consistent with the results of Experiment 1, only right-button participants showed the Simon effect in the baseline task. Most importantly, the absence of any differences between the baseline and joint tasks for both left- and right-button participants indicates that when participants perform the joint task with a co-actor who is thought to respond to their same Go color, the belief of co-acting with another individual does not exert any influence on their performance: participants behave as if they were still performing the Go/NoGo task individually.

Results of Experiment 2 are consistent with the hypothesis advanced to account for the disappearance of the Simon effect in the joint task of Experiment 1 (i.e., the division of labor hypothesis): as predicted on the basis of this hypothesis, when right-button participants were prevented from attributing the alternative response to another person, they did show a Simon effect in the joint task (as well as in the baseline task). These findings suggest that the left mouse button, which was automatically represented in the baseline task, was still active in the task representation of right-button participants during the execution of the joint task, as the color associated with this button was not attributed to any other person.

## Experiment 3

In the joint task of Experiment 1, right-button participants (just as left-button participants) were not told where the co-actor was. The absence of any information about the position of the co-actor might have caused the alternative response (the automatically represented left button press) to lose its spatial connotation when it was attributed to the co-actor. The aim of Experiment 3 was to test this hypothesis. This experiment was also aimed at evaluating the effect of the knowledge of the co-actor's position on joint-task performance of left-button participants, who did not seem to have activated any alternative response representation in either the baseline or joint task of Experiment 1.

In Experiment 3, after completing the baseline task, participants performed a joint task identical to that of Experiment 1: they were told that the co-actor was responding to the complementary color. However, unlike in Experiment 1, participants were also informed that the co-actor was acting in a room that was located on the opposite side relative to their response button (e.g., if the participant had to respond by pressing the left button, s/he was told that the co-actor was in the room on his/her right). The position of the room in which the co-actor was supposed to be always coincided with the position of the co-actor's response button (e.g., if the participant was told that the co-actor was acting in the room on the left, s/he was also told that the co-actor was responding with the left mouse button). This was meant to prevent contrasting response spatial codes: as previously discussed, there is evidence that spatial response coding can be based on different reference frames, and the Simon effect is proved to occur only when one reference frame matches the other (Dittrich et al., 2013).

The baseline task of Experiment 3 was identical to those of the previous experiments. Therefore, consistently with both Experiments 1 and 2, we expected right-button participants to show a Simon effect in the baseline task. In contrast, no Simon effect was expected for left-button participants.

Regarding to the joint task, different predictions could be made for left- and right-button participants according to whether the information about the co-actor's position has an effect on spatial response coding and depending on the type of effect. If this piece of information is sufficient either to determine the representation of the response that the co-actor is in charge of (i.e., the response alternative to the participant's response) or to make the co-actor him-/herself a reference point for spatial response coding, left-button participants should show a joint Simon effect. Conversely, if this information is not sufficient, no effect should be observed for these participants: for them we should continue to observe no differences between the two correspondence conditions, as in Experiments 1 and 2, where no information about the co-actor's position was provided.

For right-button participants, we expected that the information about the co-actor's position allowed the alternative (left) response (which is represented by virtue of practice factors) to keep its spatial connotation even when it was attributed to another person. If this were the case, the Simon effect shown by these participant in the baseline task should persist in the joint task.

### Methods

#### Participants

Sixteen undergraduate students of the University of Trento (all females; aged 19–24 years; all right-handed) participated in the experiment. They were not aware of the purpose of the experiment, did not participate in either Experiment 1 or 2 and had normal or correct-to-normal vision.

#### Apparatus, stimuli, and procedure

The apparatus, stimuli and procedure were as in the social condition of Experiment 1 with the following exceptions. Participants were told that the co-actor was performing his/her part of the task in the room spatially opposite to their response button position. The co-actor was always thought to respond to the complementary color by pressing the alternative mouse button (e.g., if the participant responded by pressing the right button, the co-actor was thought to use the left one). As a consequence, the co-actor's response button position always coincided with the position of the room in which s/he was supposed to act.

### Results and discussion

Error data (0.1%) were not analyzed. Correct mean RTs of left- and right-button participants were submitted to two ANOVAs with two within-subjects factors: task (baseline vs. critical task) and correspondence (corresponding vs. non-corresponding).

The effect of task was significant for both left and right-button participants (both *F*s ≥ 6.95, both *p*s < 0.05, both η^2^_p_s ≥ 0.50): RTs were faster in the joint than in the baseline task (312 vs. 351 ms and 322 vs. 334 ms, for left- and right-button participants, respectively).

The effect of correspondence was significant only for right-button participants, [*F*_(1, 7)_ = 18.35, *p* < 0.005; η^2^_p_ = 0.72]: responses were faster in corresponding than non-corresponding trials (322 vs. 334 ms).

**Figure 4:**
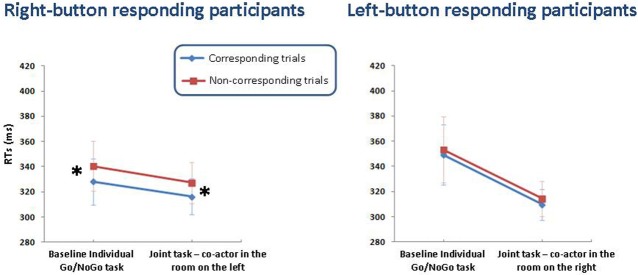
**Means (± S.E.M.s) of correct RTs for right- and left-button responding participants in Experiment 3.** RTs are plotted as a function of task (baseline vs. joint task) and spatial correspondence (corresponding vs. non-corresponding trials), separately for the two response button positions (right- vs. left-button participants). Asterisks indicate the presence of the Simon effect.

The Task × Correspondence interaction was significant for neither group of participants (see Figure 4).

The results of the baseline tasks of Experiment 3 replicate those observed in the baseline tasks of Experiments 1 and 2. The Simon effect occurred in this task only for right-button participants. Left-button participants did not show any effect.

In the joint task, left-button participants continued to show no effect. That extends the results of the joint task of Experiment 1: besides confirming that the belief of co-acting with another person—responsible for the complementary color—is not sufficient to give rise to the Simon effect when the co-actor (i.e., the alternative action) in not physically present, it demonstrates that the information about the co-actor's position is ineffective as well. Thus, even if both the co-actor's location and the location of his/her response device has been proved to be relevant for spatial response coding when the co-actor sits next to the participant (Dittrich et al., 2013), the off-line knowledge about either location, when it is sustained by no perceptual evidence (cf., Ruys and Aarts, 2010), is not able to induce the spatial coding of the participant's response ^3^. This is consistent with the idea that only a salient, dynamic event, able to attract attention, may represent an alternative action from which the participant's response has to be discriminated and thus can serve as a reference point for spatial response coding (e.g., Dolk et al., 2013a).

Important for the purpose of this experiment, in contrast to left-button participants, right-button participants showed a reliable Simon effect, not only in the baseline task, but also in the joint task. These findings are consistent with the hypothesis that the disappearance of the Simon effect observed for right-button participants in the joint task of Experiment 1 is attributable to the fact that they did not have any information about the co-actor's position. Without this piece of information, the assignment of the alternative response to the co-actor causes this response to lose its spatial connotation. Indeed, when participants know where the co-actor is, as happened in the joint task of Experiment 3, the Simon effect persists, thus suggesting that both the response associated with the complementary color and the spatial code associated to it remain active in the participants' task representation.

These findings indicate that even if the information about the co-actor's position is not sufficient to i*nduce* spatial response coding, once participant's response is spatially coded, such an information may be effective in *maintaining* the spatial coding of the response, thus contrasting the effect of a possible division of labor between the participant and the co-actor (cf., Vlainic et al., 2010).

## General discussion

The aim of the present study was to investigate the mechanisms underlying the occurrence of the interference effect in joint Simon tasks. To this end, in Experiment 1 participants performed a Go/NoGo color task first individually (baseline), and then either imaging themselves responding to the stimuli of the NoGo color (imaginative two-choice task) or co-acting with another (unseen) person, who was in a not-specified room, and was thought to respond to the NoGo stimuli (joint task). Following the most prominent interpretations of the joint Simon effect, different predictions could be made. According to the action co-representation account (see Sebanz et al., 2005, 2006; Tsai et al., 2008), knowing that the co-actor is responding to the complementary target stimuli is sufficient to activate the representation of the response associated with these stimuli. It follows that when the co-actor is thought to provide for the alternative response, the spatial coding of the participant's response and the consequent Simon effect should occur independently of the co-actor's presence in the participant's peripersonal space and regardless of the lack of information about his/her position. In contrast, according to the referential coding account (Dolk et al., 2011, 2013a), the knowledge about the co-actor's task is not necessary for the Simon effect to occur. The participant's response is assumed to be spatially coded because the co-actor constitutes a salient spatially-connoted event occurring next to the participant. As a consequence, no Simon effect should be observed when the co-actor is not physically present and executes his/her part of the task in a different room.

Participants' performance in the imaginative and joint tasks showed two different trends when compared with what was observed in the baseline task and in a control condition wherein participants continued to perform the Go/NoGo task individually. Instructions of the imaginative two-choice task made the Simon effect occur, whereas those of the joint task were ineffective in eliciting the Simon effect. Indeed, left-button participants, who did not show any effect in the baseline task, continued to show no effect in the joint task.

These findings are at odds with earlier results reported by Tsai et al. (2008) and Ruys and Aarts (2010) and indicate that, when possible confounding spatial factors are properly isolated (or controlled), the belief of co-acting with another person who is responding to the complementary color does not lead participants to represent an alternative response or, in some other way, to code their own response spatially, which is supposed to be a necessary condition for the joint Simon effect to occur. Thus, the present findings challenge the action co-representation account (Sebanz et al., 2005, 2006): they show that the information about the co-actor's task is neither necessary nor sufficient to give rise to the Simon effect.

The present results are more consistent with the referential coding account of the joint Simon effect (Dolk et al., 2011, 2013a; see also Guagnano et al., 2010 and Dittrich et al., 2012, 2013). In the light of these results, it is reasonable to reckon that the occurrence of the joint Simon effect in previous studies was due to the fact the co-actor *per se*, when acting next to the participant, represents a salient event, that is, an alternative action that participants can use as a reference to code spatially their own action. Consistently, we observed that when the co-actor is not physically present, the participant's response is not spatially coded in such a way as to produce a spatial interference effect.

On the other hand, we observed that the information about the co-actor's task is not ineffective: it can have a specific impact on participants' task representation. In the social condition of Experiment 1, indeed, the belief of co-acting with another individual, who was responding to the complementary color, made the Simon effect disappear for those participants who had shown it in the previous (baseline) task. Note that this finding, alongside with the social facilitation effect observed in all three experiments (i.e., faster RTs in the joint than in the baseline task), demonstrates the effectiveness of the social manipulation involved in our joint task, thus ensuring us that the experimental procedures used in this task make participants believe they were really sharing the task with another person. Most importantly, these findings can help us to understand the actual effect that the mere belief of co-acting with another person has on one's own task representation. They complement previous research in showing that task sharing, under some circumstances, can reduce the interference effect produced by irrelevant information compared to when people perform the task individually, rather than increase such an interference (cf., Heed et al., 2010).

As previously discussed, the Simon effect shown in the baseline task by right-button participants was probably due to the fact that these participants represented the task-irrelevant (left) button because of the familiarity with the mouse and the greater practice with this button. Task sharing caused the disappearance of an interference effect produced by the (incidental) spatial representation of an alternative response (the left response). To account for this finding, we propose that, in the baseline task, right-button participants associated the left response with the complementary color. Afterwards, since in the joint task the complementary color was up to the co-actor, participants attributed the response associated with this color, as well as the color itself, to the co-actor. Given that participants did not have any information about the co-actor's position, the response attributed to the co-actor might have lost its spatial connotation, which resulted in the disappearance of the Simon effect. In the baseline task, the response of these participants was coded as on the right because it was on the right with respect to the other, non-requested but still represented, response. When this spatial reference point was no longer available, no spatial response coding, and thus no matches/mismatches between stimulus and response codes occurred that could give rise to the Simon effect.

This hypothesis is supported by the results of Experiments 2 and 3: the Simon effect shown by right-button participants in the baseline task continued to occur in the joint task both when the co-actor was thought to perform the same task as the participant (i.e., the participant and the co-actor responded to the same color; Experiment 2) and even if the co-actor was thought to respond to the complementary color (as in Experiment 1) as long as participants knew where the co-actor was (Experiment 3). The persistence of the Simon effect in the joint task of Experiment 2 is consistent with the hypothesis that when the co-actor works on the same stimuli as the participant, the alternative response cannot be attributed to anyone else and, consequently, it remains active in participants' task representation and can keep serving as a reference for spatial response coding. Conversely, the persistence of the Simon effect in the joint task of Experiment 3 supports the hypothesis that when the spatial information about the co-actor's position is provided, this kind of information allows the alternative response to keep its spatial connotation, even if attributed to another person.

It is worth noting that, even when the position of a person is known, the mere belief of co-acting with him/her does not seem to induce spatial response coding so as to cause interference effects, as indicated by the fact that left-button participants never showed a Simon effect in the joint task, not even in Experiment 3. The knowledge about the co-actor's position only had an effect for right-button participants who had already (and irrespective of this knowledge) represented two distinct spatial responses. Indeed, spatial effects observed in the present study can only be ascribed to the representation of the alternative response of the participants themselves, because of either the familiarity with the alternative button (in the right-button condition) or the explicit request of the instructions (in the imaginative two-choice task). Neither the position of the co-actor's response, nor the position of the co-actor himself/herself seems to have been represented or used as a reference for spatial response coding. The knowledge about the position of the co-actor only prevented the alternative response of the participant, which was represented by virtue of other, non-social factors, from losing its spatial connotation. Here, indeed, given that no perceivable feedback came from the co-actor, who was not physically present and was thought to perform his/her task relatively far from the participant, the co-actor and his/her response did not represent a salient, task-relevant event from which the participant response has to be discriminated and cannot serve as reference for spatial coding (see Guagnano et al., 2010; Dolk et al., 2011, 2013a).

Such considerations can help us in shedding light on the results of previous studies that used paradigms similar to that employed here. In the joint task of Tsai et al. (2008), a significant Simon effect was observed. As previously underlined, participants in this study responded with the right mouse button (i.e., Tsai et al.'s task was comparable to our joint right-button task) but no baseline data were collected, thus we cannot evaluate the actual effect of the social manipulation: we do not know how large the Simon effect would be in a (hypothetical) individual condition and whether the social manipulation increases or decreases such an effect. Nevertheless, it is worth underlining another aspect which may have contributed to the occurrence of the Simon effect (or which have prevented its disappearance despite the division of labor), that is, the fact that Tsai et al.'s participants knew the position of the room in which the co-actor was acting, which would render Tsai et al.'s task similar to the joint task of our Experiment 3.

On the whole, the results of this study make a substantial contribute to the comprehension of the mechanisms underlying the joint Simon effect. They are consistent with the referential coding account in suggesting that social factors (the mere knowledge of the co-actors' task when the co-actor is thought to perform the complementary task) are neither necessary nor sufficient for the occurrence of the joint Simon effect (unlike what the action co-representation account proposes). Furthermore, they demonstrate that the information about the co-actor's task can influence participants' performance both by speeding up participants' RTs and by causing the disappearance of the interference effect produced by the (incidental) spatial representation of an alternative response.

Further studies are needed to understand better the mechanisms underlying the disappearance of spatial interference effects when participants share their task with other people. On the basis of the available evidence, we can conclude that the participant–co-actor division of labor is not effective in eliminating the Simon effect when the co-actor's position is known and, all the more so, when the co-actor's position is perceptually evident, as in the standard joint Simon task, where the co-actor sits next to the participant. In the latter case, indeed, the co-actor cannot serve as a ploy to filter out the irrelevant alternative response, thus preventing the potentially interfering spatial response coding, but rather is him/herself the reference point for the spatial coding of the participant's response.

However, many questions remain open about this issue. Since the pivotal study of Sebanz et al. (2003), researchers have wondered “How «social» is the social Simon effect” (cf., Dolk et al., 2011), which can be put into the question of whether the joint Simon effect depends on the representation of the co-actor's aims and intentions. Although the joint Simon effect has been observed even when the action alternative to the participant's response is provided by a non-human agent that works in a purely deterministic machine-like manner (i.e., a metronome; Dolk et al., 2013a), there is evidence that, when the co-actor is an artificial agent (i.e., a robot or a puppet), the magnitude of the effect can be modulated by the intentionality attributed to the co-actor (Müller et al., 2011; Stenzel et al., 2012). These findings have been accounted for by assuming that the degree of task co-representation may vary and depends on how much the co-actor is perceived as functioning in a human-like or biological inspired way (Stenzel et al., 2012). However, they can also be accounted for by the referential coding account by assuming that the more similar the action events referring to the participant and co-actor are, the more difficult the discrimination between them is, which, in turn, makes their relative spatial coding more necessary (Dolk et al., 2013a).

As with the mechanisms underlying the occurrence of the Simon effect in joint tasks, one may wonder how social is the nature of the mechanisms underlying its disappearance in task-sharing contexts. In the joint task of Experiment 1, right-button participants showed to be able to filter out the response associated with the NoGo stimuli irrespective of the specific action executed by the co-actor to take care of these stimuli (i.e., the button pressed by the co-actor). This suggests that the co-actor may simply provide participants with an effective strategy to ignore the irrelevant stimuli and their associated response and that the effectiveness of this strategy does not crucially rely on the representation of what the co-actor specifically intends to do. Nevertheless, further studies should be conducted in order to test whether such a division-of-labor strategy depends on the co-actor being perceived as an agent with intentional behaviors.

## Funding

This study was supported by the Provincia autonoma di Trento, the Fondazione Cassa di Risparmio di Trento e Rovereto (Italy), the Dipartimento di Storia, Scienze dell'Uomo e della Formazione (University of Sassari), and University of Modena and Reggio Emilia.

### Conflict of interest statement

The authors declare that the research was conducted in the absence of any commercial or financial relationships that could be construed as a potential conflict of interest.

## Footnotes

^1^As recently shown, participants can easily imagine themselves performing a response they do not actually execute provided that the response device is present and they have the possibility to operate it (i.e., they are not prevented from reaching it; Iani et al., 2013).

^2^Another possible explanation of the slower RTs in the imaginative task compared to the joint task is that the instructions of the imaginative task (just as those of a standard two-choice Simon task) induced an extra working memory load (participants had to keep in memory both complementary stimulus-response mappings; e.g., red target–left button press and green target–right button press), which makes this task more demanding. However, the occurrence in this task of a sizeable Simon effect, comparable to the standard effect (see below in the main text), corroborates the hypothesis that two spatially alternative responses were represented in a similar fashion to what happens in standard two-choice tasks.

^3^It has been proposed that another potential source of interference in joint tasks is the conflict related to agent identification (i.e., determining whether it is “my” or the other's turn; Wenke et al., 2011). Participants in joint task settings would represent that another person is responsible for the complementary task, and when the other person has to respond (i.e., the stimulus conditions under which it is the co-actor's turn). Participants would use all available cues to distinguish between their own turn and the co-actor's turn. In joint Simon tasks, participants would use the stimulus position: the stimulus position is seen as signaling the co-actor's turn when it corresponds to the co-actor's position, thus slowing RTs in noncorresponding trials. On the basis of the results of the present study we cannot rule out that this type of interference has a role in standard joint tasks, wherein the co-actor sits next to the participant and the stimulus positions strictly coincides with the participant's and co-actor's (absolute) positions. In this kind of task, the stimulus can be seen as pointing to either the participant or the co-actor. However, the absence of spatial effects for left-button participants in both Experiments 1 and 3 show that people do not use relative spatial correspondences as turn-taking signals: neither the correspondence of the stimulus position with the position of the co-actor's response relative to the participant's response nor the correspondence of the stimulus position with the position of the co-actor's room relative to the participant's room are used as an indication of the co-actor's turn.
